# Spiro annulation of cage polycycles via Grignard reaction and ring-closing metathesis as key steps

**DOI:** 10.3762/bjoc.11.147

**Published:** 2015-08-05

**Authors:** Sambasivarao Kotha, Mohammad Saifuddin, Rashid Ali, Gaddamedi Sreevani

**Affiliations:** 1 Department of Chemistry, Indian Institute of Technology-Bombay, Powai, Mumbai-400 076, India, Phone: +91-22-2576 7160, Fax: +91(22)-2572 7152

**Keywords:** cage molecules, Diels–Alder reaction, Grignard reaction, ring-closing metathesis, spirocycles

## Abstract

A simple synthetic strategy to *C*_2_-symmetric bis-spiro-pyrano cage compound **7** involving ring-closing metathesis is reported. The hexacyclic dione **10** was prepared from simple and readily available starting materials such as 1,4-naphthoquinone and cyclopentadiene. The synthesis of an unprecedented octacyclic cage compound through intramolecular Diels–Alder (DA) reaction as a key step is described. The structures of three new cage compounds **7**, **12** and **18** were confirmed by single crystal X-ray diffraction studies.

## Introduction

Design and synthesis of architecturally intricate cage molecules is a worthwhile challenge. The unique properties associated with the carbocyclic cage frameworks are the main reasons for pursuing their synthesis [1–2]. They are valuable synthons to assemble natural as well as non-natural products [3–4]. In addition, the cage molecules are interesting targets because of their unusual structural features such as the deformation of the ideal C–C bond angles, high degree of symmetry and the enhanced ring strain etc. [5–18].

**Figure 1:**
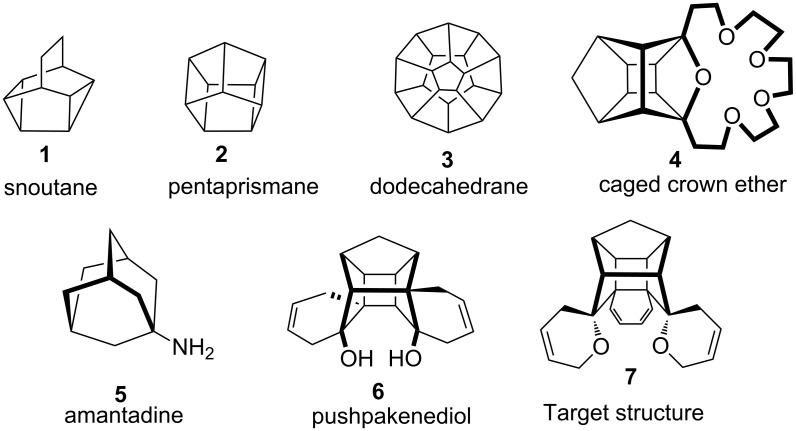
Structures of diverse biologically as well as theoretically interesting molecules.

The structures of a variety of intricate cage systems, for example, snoutane (**1**) [5], pentaprismane (**2**) [10], dodecahedrane (**3**) [11–19], cage crown ether **4** [20], amantadine (**5**) and pushpakenediol (**6**) [21] along with the target molecule **7** are shown in Figure 1. Interestingly the amino group containing cage molecule amantadine (**5**) exhibits antiviral properties [22].

**Figure 2:**
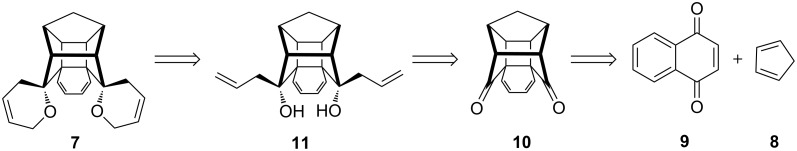
Retrosynthetic analysis of bis-spiro-pyrano cage compound **7**.

Although, several methods are available for the construction of cage compounds [7,23–33], the synthesis of symmetrical spiro-cage molecule **7** seems to be a synthetic challenge due to the proximity of the two carbonyl groups in dione **10** which provides a hemiketal with various nucleophiles [34–39]. In view of various applications of cage molecules and the documented difficulties in their synthesis, we conceived a short synthetic route to *C*_2_-symmetric bis-spiro-pyrano cage compound **7**. To this end, the Grignard addition and ring-closing metathesis (RCM) are considered as viable options. The retrosynthetic analysis to the target bis-spiro-cage compound **7** is shown in Figure 2. The target compound **7** could be obtained from *O*-allylation of the Grignard addition product **11** followed by the two-fold RCM sequence. The required cage dione **10** could be constructed in two steps from readily available starting materials such as 1,4-naphthoquinone (**9**) and cyclopentadiene (**8**) [40–41].

## Results and Discussion

**Scheme 1:**
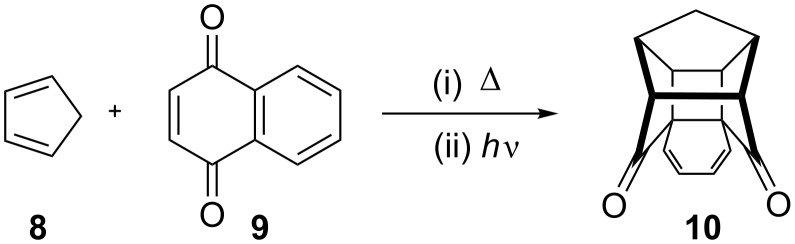
Synthesis of hexacyclic cage dione **10**.

In connection with the synthesis of new cage molecules, we reported a new approach to the hexacyclic dione **10** and related systems via Claisen rearrangement and RCM as key steps [21,30]. Here, we have prepared the cage dione **10** by the known route involving two atom-economic protocols such as Diels–Alder reaction and [2 + 2] photocycloaddition [42–45] (Scheme 1).

**Scheme 2:**
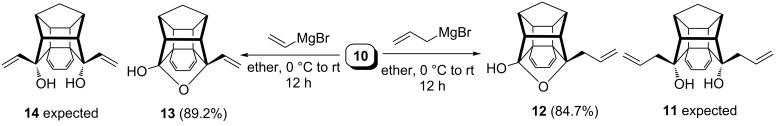
Synthesis of tetrahydrofuran-based cage compounds **12** and **13**.

Later, the hexacyclic cage dione **10** was subjected to a Grignard reaction with comercially available allylmagnesium bromide in diethyl ether. Under these conditions, we realized the formation of hemiketal **12** in 84.7% yield instead of the expected diallylated product **11** (Scheme 2). In similar fashion, the cage dione **10** was treated with comercially available vinylmagnesium bromide and the hemiketal **13** [46–47] was obtained in 89.2% yield instead of the desired divinylated compound **14** (Scheme 2). The proximity of the carbonyl groups may be responsible for the formation of hemiketals.

**Figure 3:**
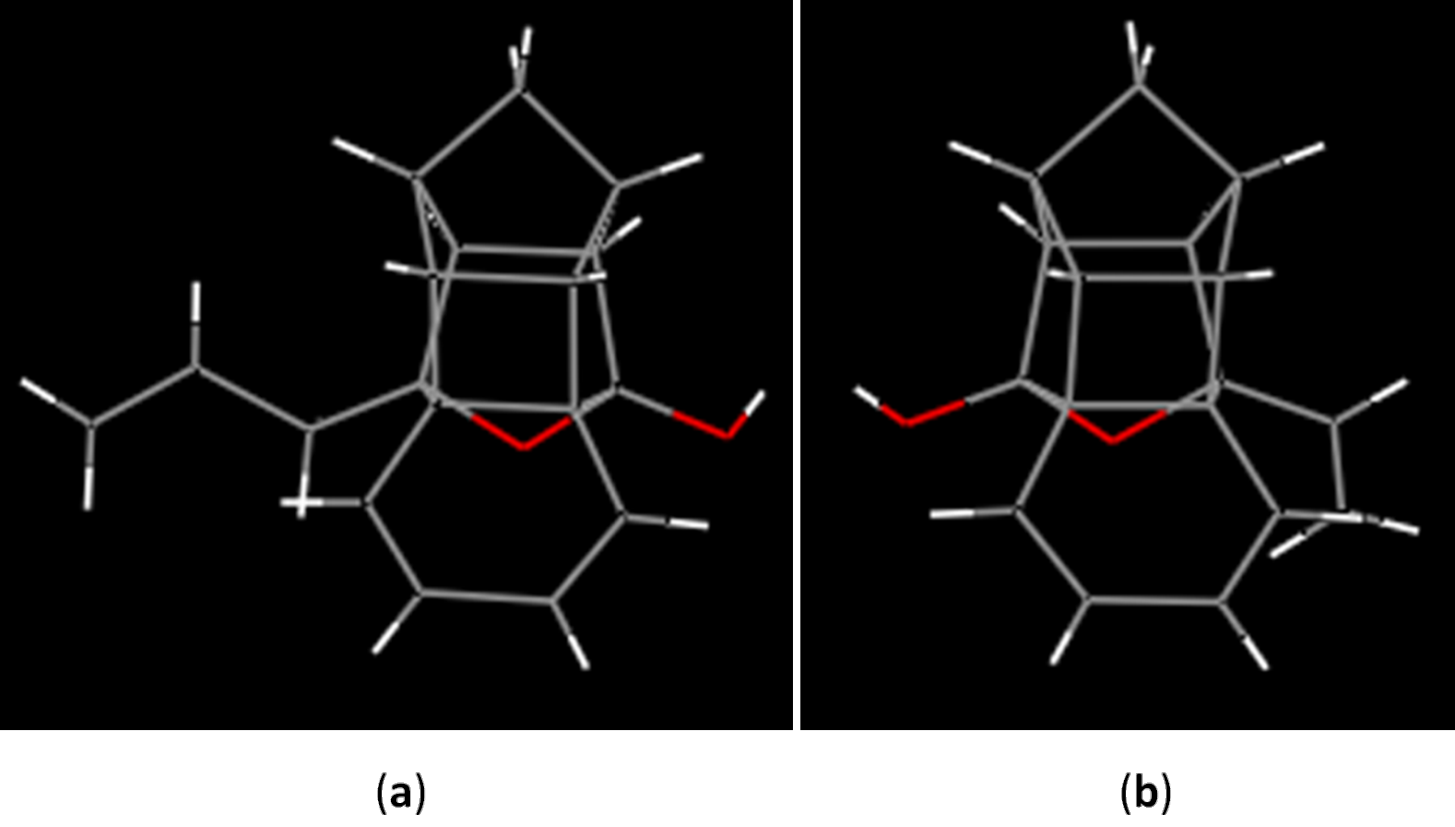
(a)Optimized structure of **12**, (b) optimized structure of **13**.

The structures of both these heptacyclic hemiketals **12** and **13** have been confirmed by ^1^H and ^13^C NMR spectral data and further supported by HRMS data. Finally their structures have been unambiguousily established by single crystal X-ray diffraction studies [48] (Figure 3).

**Scheme 3:**
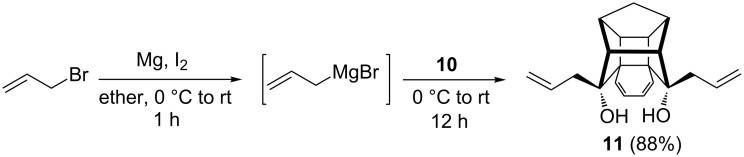
Synthesis di-allyl cage compound **11**.

Since our goal was to synthesize the diallylated compound **11**, we screened various reaction conditions and finally, we found that the addition of the etheral solution of the hexacyclic dione **10** to a freshly prepared allyl Grignard reagent at 0 °C gave the expected diallylated compound **11** in 88% yield (Scheme 3). The Grignard reagent at higher concentration (1.0 M solution) exists as a mixture of dimer, trimer and polymeric components. However, the home-made Grignard reagent at low concentration (0.1 M solution) exists mostly in the monomeric form. So, we speculate that the difference in the concentration may be responsible for the formation of diol **11** [49–51]. Alternatively, when the diketone was reacted with an excess amount of Grignard reagent, the carbonyl groups are attacked simultaneously by the Grignard reagent and resulted in the formation of diol **11**. When an excess amount of substrate containing carbonyl group was reacted with a limited amount of Grignard reagent, the oxyanion formed by the Grignard reagent attacks the other carbonyl group in a transannular fashion to generate hemiketal derivatives **12** and **13**.

**Scheme 4:**
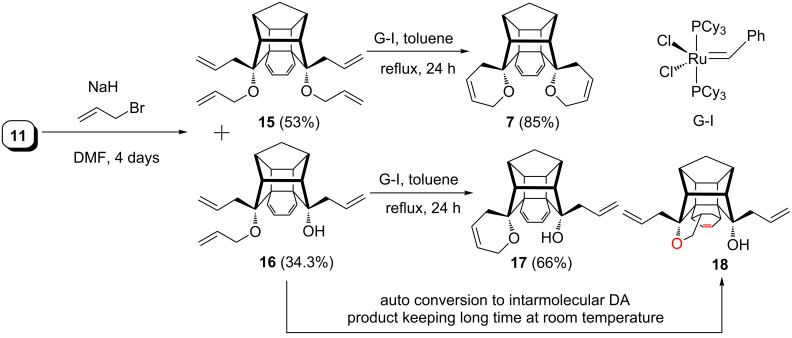
Synthesis of spiro-pyrano cage molecules **7** and **17**.

Later, the diallyldiol **11** was subjected to an *O*-allylation sequence under NaH/allyl bromide conditions in DMF to deliver the desired tetraallyl compound **15** (53%) along with the triallyl compound **16** (34.3%) (Scheme 4). Subsequently, the tetraallyl compound **15** was subjected to an RCM sequence with the aid of Grubbs’ first generation catalyst (G-I) in dry CH_2_Cl_2_. Surprisingly under these conditions the reaction was found to be sluggish.

Therefore, various other reaction conditions were screened to optimize the yields. Finally, we found that the Grubbs’ first generation catalyst (G-I) in refluxing toluene gave the desired RCM product **7** in 85% yield. Along similar lines, the triallyl compound **16** gave the RCM product **17** in 66% yield (Scheme 4).

**Figure 4:**
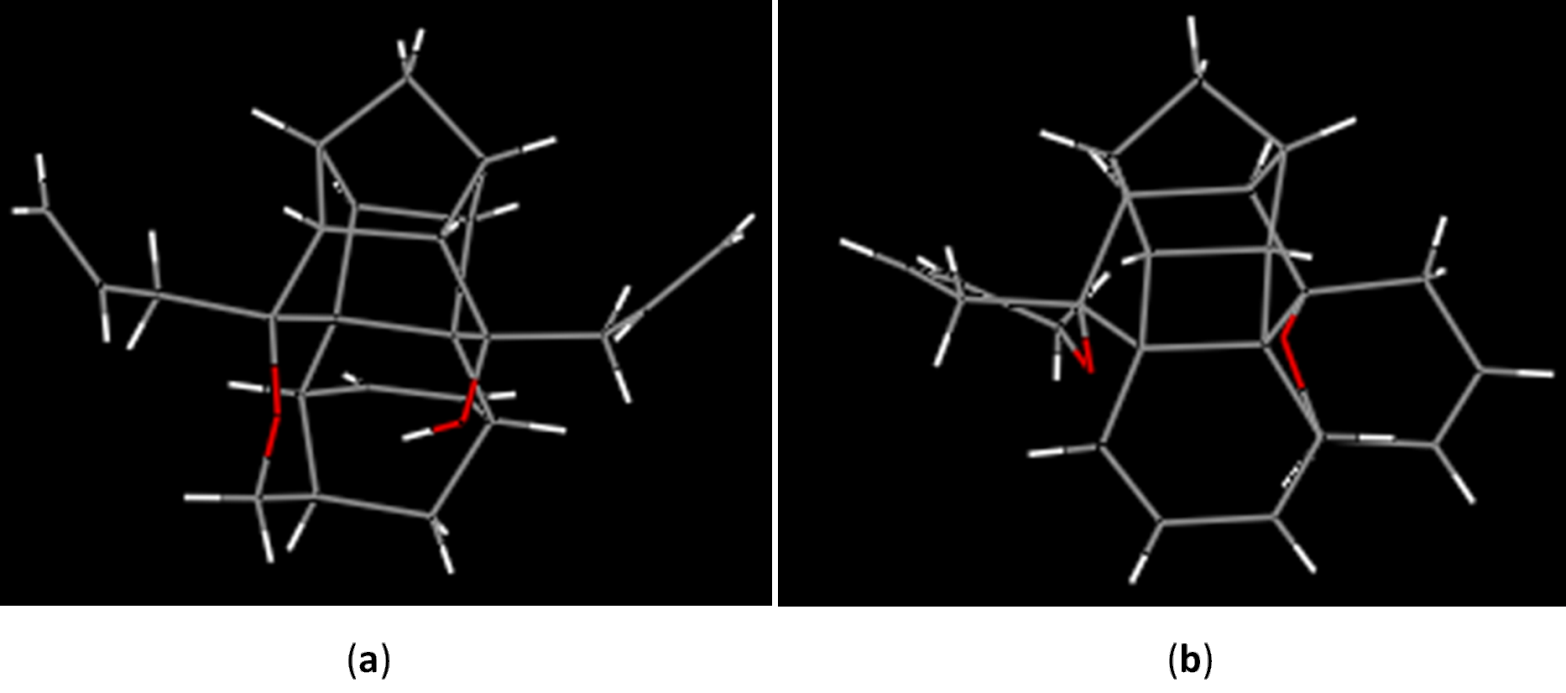
(a) Optimized structure of **18**, (b) optimized structure of **7**.

The structures of the annulated cage compounds **7** and **17** have been confirmed by ^1^H and ^13^C NMR spectral data and also supported by HRMS data with a molecular weight of 355.16 for **7** and 343.16 for compound **17**, respectively. Furthermore, the structure of the bis-spiro pyrano cage compound **7** was confirmed by single crystal X-ray diffraction studies [52] (Figure 4). Fortunately, we observed that the liquid compound **16** kept at room temperature for a long time converted into a solid material. Therefore, we were keen to investigate the reason for this observation. In this context, the ^1^H and ^13^C NMR spectra of this compound were again recorded, indicating the occurence of an intramolecular DA reaction. Later, it was confirmed by single crystal X-ray diffraction studies [53] (Figure 4).

**Scheme 5:**
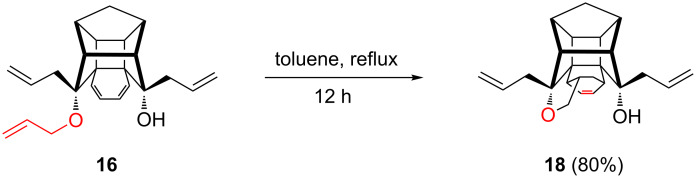
Synthesis of octacyclic cage compound **18** via intramolecular DA reaction.

Next, the formation of compound **18** has been confirmed by an independent synthesis. To this end, triallyl compound **16** was subjected to intramolecular DA reaction in refluxing toluene to deliver the DA adduct **18** in 80% yield (Scheme 5).

**Scheme 6:**
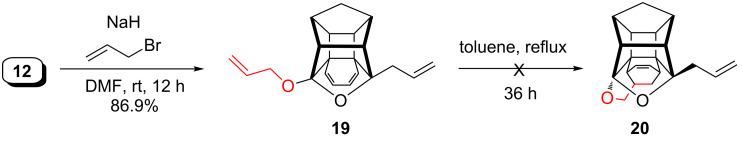
Attempted synthesis to cage compound **20**.

Surprisingly the related system **19**, prepared from **12** did not undergo DA reaction to produce the intramolecular DA adduct **20**. Even under prolonged toluene reflux reaction conditions, we did not realize the formation of the required DA product **20** (Scheme 6).

## Conclusion

In summary, we have demonstrated a new approach to intricate *C*_2_-symmetric cage bis-spirocyclic pyran derivative **7** through an allyl Grignard reaction and an RCM sequence. The strategy demonstrated here involves an atom economic process. The synthetic sequence demonstrated here opens up a new route to complex cage targets. Additionally, intramolecular DA reaction opens up a new strategy for the synthesis of highly complex cage compounds that are inaccessible by other routes. Studies to extend the scope of the intramolecular as well as intermolecular DA reaction for the synthesis of interesting cage molecules are in progress.

## Supporting Information

File 1 Detailed experimental procedures, characterization data and copies of ^1^H and ^13^C NMR spectra for all new compounds.
