# Supplementary material for: Spiro annulation of cage polycycles via Grignard reaction and ring-closing metathesis as key steps
Source: Beilstein J Org Chem. 2015 Aug 5;11:1367–72. doi: 10.3762/bjoc.11.147 (PMC4578432; doi:10.3762/bjoc.11.147)
Supplement: File 1 — Detailed experimental procedures, characterization data and copies of 1H and 13C NMR spectra for all new compounds. [file Beilstein_J_Org_Chem-11-1367-s001.pdf]

Supporting Information

for

Spiro annulation of cage polycycles via Grignard reaction and ring-closing metathesis as key steps

Sambasivarao Kotha*, Mohammad Saifuddin, Rashid Ali and Gaddamedi Sreevani

Address: Department of Chemistry, Indian Institute of Technology-Bombay, Powai, Mumbai-400 076, India, Phone: +91-22-2576 7160, Fax: +91(22)-2572 7152

Email: Sambasivarao Kotha - srk@chem.iitb.ac.in

*Corresponding author

Detailed experimental procedures, characterization data and copies of ^{1}H and ^{13}C NMR spectra for all new compounds.

Table of contents

General information and methods S2

General procedure and characterization of compounds 7, 11, 12, 13, 15, 16, 17, 18, and 19 S3

Copies of ^{1}H and ^{13}C NMR spectra 7, 12, 13, 15, 16, 17, 18, 19, and X-ray data S8

General information and methods

All commercially accessible reagents were used without further purification. Reactions involving air sensitive catalysts or reagents were performed in degassed solvents. Moisture sensitive materials were transferred using syringe-septum techniques and the reactions were maintained under nitrogen atmosphere. Analytical thin layer chromatography (TLC) was performed on (7.5 × 2.5 cm) glass plates coated with Acme's silica gel GF 254 (containing 13% calcium sulfate as a binder) by using a suitable mixture of EtOAc and petroleum ether for the development. Column chromatography was performed by using Acme's silica gel (100–200 mesh) with an appropriate mixture of EtOAc and petroleum ether. The coupling constants (*J*) are given in hertz (Hz) and chemical shifts are denoted in parts per million (ppm) downfield from the internal standard tetramethylsilane (TMS). The abbreviations, s, d, t, q, m, dd and td, refer to singlet, doublet, triplet, quartet, multiplet, doublet of doublets, and triplet of doublets respectively. Grubbs catalysts were purchased from Sigma Aldrich. Infrared (IR) spectra were recorded on a Nicolet Impact-400 FT IR spectrometer in $CHCl_3$. Proton nuclear magnetic resonance (^{1}H NMR, 400 MHz and 500 MHz) spectra and carbon nuclear magnetic resonance (^{13}C NMR, 100 MHz and 125 MHz) spectra were recorded on a Bruker spectrometer. The high-resolution mass measurements were carried out by using an electrospray ionization (ESI, Q-ToF) spectrometer. Melting points were recorded on a Veego melting point apparatus.

Compound **10** was prepared according to a literature procedure[1]

[1] (a). Kushner, A. S. *Tetrahedron Lett*. **1971**, *35*, 3275. doi:10.1016/S0040-4039(01)97154-0. (b). Valiulin, R. A.; Arisco, T. M.; Kutateladze, A. G. *J. Org. Chem.* **2011**, *76*, 1319. doi: 10.1021/jo102221q

Synthesis of compound 12 via Grignard reaction

To a solution of compound **10** (100 mg, 0.44 mmol) in dry ether (10 mL), was added commercially available allylmagnesium bromide (1.0 M in THF, 6 equiv) at 0 °C dropwise and the reaction mixture was stirred at rt for 12 h. After completion of the reaction (TLC monitoring), the reaction mixture was quenched with saturated aq. NH_4Cl solution (5 mL) and the aqueous layer was extracted with EtOAc (3 × 10 mL). The solvent was removed under reduced pressure and the crude product was purified by column chromatography (100–200 silica gel) using appropriate mixtures of EtOAc–petroleum ether as an eluent to afford the desired compound **12** (100 mg, 84.7%).

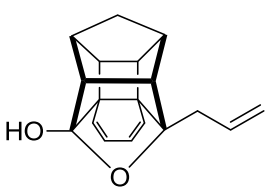

White solid, mp = 128–130 °C, IR (Neat) ν_{max} 3363, 2941, 1639, 1449, 1324, 1149, 1136, 912 cm^{-1}; ^{1}H NMR (400 MHz, $CDCl_3$) δ 1.37 (d, *J* = 10.6 Hz, 1H), 1.75-1.79 (m, 1H), 2.35-2.39 (m, 1H), 2.44-2.47 (m, 1H), 2.52-2.56 (m, 1H), 2.64-2.65 (m, 1H), 2.71-2.72 (m, 1H), 2.77-2.80 (m, 2H), 2.89-2.91 (m, 1H), 3.45 (s, 1H), 5.10-5.18 (m, 2H), 5.48-5.51 (m, 2H), 5.80-5.90 (m, 3H); ^{13}C NMR (100 MHz, $CDCl_3$) δ 34.5, 41.9, 43.1, 44.3, 52.7, 54.4, 54.6, 54.8, 57.9, 58.3, 94.2, 118.2, 118.5, 120.3, 122.8, 123.9, 125.0, 133.7 ppm; HRMS (ESI) calcd for $C_{18}H_{18}NaO_2$[M + Na] 289.1199 found 289.1195.

Synthesis of compound 13 *via* Grignard reaction

To a solution of compound **10** (50 mg, 0.22 mmol) in dry ether (10 mL), was added commercially available vinylmagnesium bromide (1.0 M in THF, 6 equiv) at 0 °C dropwise and the reaction mixture was stirred at rt for 12 h. After completion of the reaction (TLC monitoring), the reaction mixture was quenched with saturated aq. NH_4Cl solution (5 mL) and the aqueous layer was extracted with EtOAc (3 × 10 ml). The solvent was removed under reduced pressure and the crude product was purified by column chromatography (100–200 silica gel) using appropriate mixtures of EtOAc–petroleum ether as an eluent to afford the desired compound **13** (50 mg, 89.2%).

White solid, mp = 102–103 °C (lit.[2] mp 111–112 °C), IR (Neat) ν_{max} 3362, 2936, 1640, 1432, 1345, 1322, 1149, 1134, 1025, 910 cm^{-1}; ^{1}H NMR (500 MHz, $CDCl_3$) δ 1.40 (d, *J* = 10.5 Hz, 1H), 1.80 (d, *J* = 10.5 Hz, 1H), 2.59-2.65 (m, 1H), 2.66-2.67 (m, 1H), 2.74-2.77 (m,

[2]. Bott, S. G.; Marchand, A. P.; Kumar, K. A. *J. Chem. Crystallogr*. **1996**, *26*, 429-433. doi: 10.1007/BF01665824

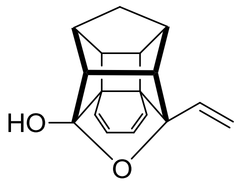

1H), 2.895-2.898 (m, 2H), 2.90-2.92 (m, 1H), 5.22-5.28 (m, 2H), 5.37 (d, J = 10.0 Hz, 1H), 5.52 (d, J = 9.5 Hz, 1H), 5.75-5.78 (m, 1H), 5.84-5.87 (m, 1H), 5.93-5.98 (m, 1H); ^{13}C NMR (125 MHz, $CDCl_3$) δ 41.9, 42.8, 44.8, 52.4, 54.4, 54.5, 56.8, 58.1, 59.3, 95.0, 115.7, 118.9, 120.3, 123.2, 123.4, 124.9, 113.9 ppm; HRMS (ESI) calcd for $C_{17}H_{16}NaO_2$[M + Na] 275.1043 found 275.1044.

Synthesis of compound 11 via Grignard reaction

To a freshly prepared solution of allylmagnesium bromide (6.0 equiv) in ether at 0 °C was added an etheral solution of compound **10** (50 mg, 0.22 mmol) in a dropwise manner and the reaction mixture was stirred at 0 °C for 1 h and then at rt for further 12 h. After completion of the reaction as evident by TLC analysis, the reaction mixture was quenched with saturated aq. NH_4Cl solution (5 mL) and the aqueous layer was extracted with EtOAc (3 × 10 mL). The organic layer was washed with brine, dried over Na_2SO_4 and concentrated in vacuo. The crude product was purified by column chromatography (100–200 silica gel) using appropriate mixtures of EtOAc–petroleum ether as eluent to afford the desired compound **11** (60 mg, 88.2%). (Respective data were identical to those from the previous publication) [3]

Synthesis of compounds 15 and 16

To a suspension of sodium hydride (6.0 equiv) in dry DMF (15 mL), was added the compound **11** (60 mg, 0.19 mmol) and the reaction mixture was stirred for 30 min at rt. Allyl bromide (6 equiv) was then added and stirring was continued for 4 days at the same temperature. After completion of the reaction (TLC monitoring), the reaction mixture was diluted with saturated aq. NH_4Cl (5 mL) and aqueous layer was then extracted with EtOAc (3 × 10 mL). The organic layer was washed with brine and dried over Na_2SO_4. The solvent was removed under reduced pressure and the crude product was purified by column chromatography (100–200 silica gel) using appropriate mixtures of EtOAc–petroleum ether as eluent to afford the desired compounds **15** (40 mg, 53 %) and **16** (23 mg, 34.3%).

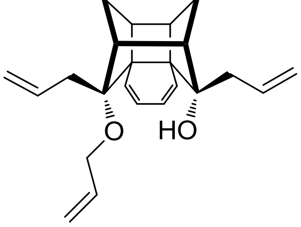

Spectral data of compound 16

White liquid, IR (Neat) ν_{max} 3365, 2949, 2938, 2864, 1656, 1218, 1047 cm^{-1}; ^{1}H NMR (500 MHz, $CDCl_3$) δ 0.88 (d, J = 13.5 Hz, 1H), 1.39 (d, J = 13.5 Hz, 1H), 1.86-1.95 (m, 2H), 2.23-2.25 (m, 1H),

[3]. Kotha, S.; Dipak, M. K. Beilstein *J. Org. Chem.* **2014**, *10*, 2664. doi: 10.3762/bjoc.10.280

2.35-2.38 (m, 1H), 2.40-2.51 (m, 3H), 2.63-2.67 (m, 1H), 2.75-2.84 (m, 2H), 4.13 (d, J = 6.9 Hz, 2H), 5.07-5.16 (m, 5H), 5.26 (dd, J_1 =21.4, J_2 = 1.8 Hz, 1H), 5.62-5.70 (m, 2H), 5.79-5.99 (m, 4H), 6.00-6.08 (m, 1H), 6.87 (d, J = 3.1 Hz, 1H); ^{13}C NMR (125 MHz, $CDCl_3$) δ 31.5, 34.8, 42.0, 43.5, 43.8, 49.0, 49.1, 49.7, 51.5, 55.0, 55.5, 65.7, 78.5, 84.3, 116.9, 117.8, 118.0, 122.5, 123.7, 125.0, 125.7, 132.9, 133.6, 134.9 ppm; HRMS (ESI) calcd for $C_{24}H_{28}NaO_2$[M + Na] 371.1982 found 371.1983.

Spectral data of compound 15

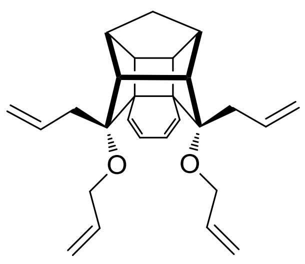

White solid, mp. = 115–117 °C, IR (Neat) ν_{max} 3417, 3152, 2938, 1456 cm^{-1}; ^{1}H NMR (500 MHz, $CDCl_3$) δ 0.89 (d, J = 10.9 Hz, 1H), 1.38 (d, J = 10.8 Hz, 1H), 2.04 (dd, J_1 = 14.9, J_2 = 7.9 Hz, 2H), 2.32 (d, J = 1.7 Hz, 2H), 2.41 (d, J = 1.4 Hz, 2H), 2.60 (dd, J_1 = 14.9, J_2 = 6.0 Hz, 2H), 2.66 (d, J = 2.5 Hz, 2H), 4.02-4.11 (m, 4H), 4.96-4.99 (m, 2H), 5.05-5.16 (m, 6H), 5.71-5.74 (m, 2H), 5.78-5.87 (m, 4H), 5.99-6.07 (m, 2H); ^{13}C NMR (125 MHz, $CDCl_3$) δ 31.3, 40.0, 44.3, 48.7, 50.9, 55.5, 66.2, 82.9, 114.8, 116.7, 122.1, 126.3, 135.6, 137.2 ppm; HRMS (ESI) calcd for $C_{27}H_{32}NaO_2$[M + Na] 411.2295 found 411.2303.

General procedure for the synthesis of compounds 17 and 7 via RCM sequence

To a stirred solution of compounds **16** (23 mg, 0.07 mmol) and **15** (40 mg, 0.11 mmol) in dry toluene (20 mL) degassed with nitrogen for 15 min was added G-I (10 mol %) catalyst and the reaction mixture was stirred under reflux for 12 h. After completion of the reaction (TLC monitoring), the solvent was removed under reduced pressure and the crude products were purified by column chromatography (100–200 silica gel) using appropriate mixtures of EtOAc-petroleum ether as eluent to afford the RCM products **17** (14 mg, 66%) and **7** (29 mg, 85%) respectively.

Spectral data of compound 17

White liquid, IR (Neat) ν_{max} 3439, 2959, 2931, 2873, 2864, cm^{-1}; ^{1}H NMR (500 MHz, $CDCl_3$) δ 0.93 (d, J = 10.9 Hz, 1H), 1.42 (d, J = 10.8 Hz, 1H), 1.94-1.98 (m, 1H), 2.05 (d, J = 18.5 Hz, 1H), 2.15-2.30 (m, 3H), 2.43-2.49 (m, 3H), 2.65-2.77 (m, 2H), 4.11 (d, J = 16.1 Hz, 1H), 4.44 (d, J = 16.0 Hz, 1H), 5.12 (d, J = 13.3 Hz, 2H), 5.63-5.73 (m, 4H), 5.81-5.84 (m, 1H), 5.88-5.91 (m, 1H), 6.02-6.07 (m, 1H), 7.35 (d, J = 2.2 Hz, 1H); ^{13}C NMR (125 MHz,

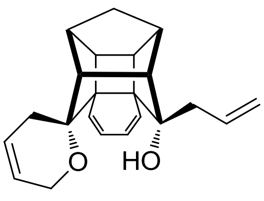

$CDCl_3$) δ 31.6, 33.1, 41.9, 43.3, 43.4, 49.6, 49.7, 51.7, 52.8, 54.4, 55.1, 63.4, 78.5, 80.2, 117.0, 121.8, 123.7, 124.3, 124.9, 125.3, 126.2, 134.7 ppm; HRMS (ESI) calcd for $C_{22}H_{24}NaO_2$[M + Na] 343.1669 found 343.1662.

Spectral data of compound 7

White solid, mp = 106–108 °C, IR (Neat) ν_{max} 2952, 2932, 2877, 1463, 1274 cm^{-1}; ^1H NMR

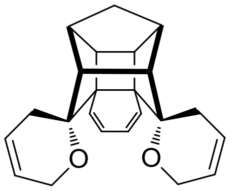

(400 MHz, $CDCl_3$) δ 0.89-0.92 (m, 1H), 1.40 (d, J = 10.8 Hz, 1H), 1.98-2.05 (m, 2H), 2.10-2.16 (m, 2H), 2.33 (d, J = 1.8 Hz, 2H), 2.41-2.44 (m, 2H), 2.67 (d, J = 2.6 Hz, 2H), 4.07-4.14 (m, 2H),4.22-4.29 (m, 2H), 5.71-5.81 (m, 8H); ^{13}C NMR (100 MHz, $CDCl_3$) δ 31.4, 33.3, 43.8, 49.9, 50.7, 55.5, 63.9, 79.7, 121.5, 124.3, 126.9, 127.4 ppm; HRMS (ESI) calcd for $C_{23}H_{24}NaO_2$[M + Na] 355.1669 found 355.1665

Synthesis of compound 18 *via* intramolecular Diels–Alder reaction

The compound **16** (15 mg) in dry toluene (20 mL) under N_2 atmosphere was stirred at reflux for 12 h. After completion of the reaction (TLC monitoring), the solvent was removed under reduced pressure and the crude products were purified by column chromatography (100–200 silica gel) using appropriate mixtures of EtOAc–petroleum ether as eluent to afford the DA product **18** (12 mg, 80%).

White solid, mp. = 167–168 °C, IR (Neat) ν_{max} 3342, 3020, 2943, 1642 cm^{-1}; ^1H NMR (500

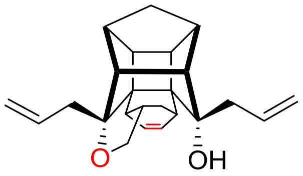

MHz, $CDCl_3$) δ 1.01 (d, J = 10.6 Hz, 1H), 1.28 (td, J_1 = 12.6, J_2 = 2.1 Hz, 1H), 1.43 (d, J = 10.6 Hz, 1H), 1.68-1.77 (m, 2H), 1.87-1.92 (m, 2H), 2.10-2.14 (m, 2H), 2.19-2.21 (m, 1H), 2.33-2.35 (m, 1H), 2.44-2.48 (m, 2H), 2.55 (dt, J_1 = 5.5, J_2 = 2.8 Hz, 1H), 2.65-2.71 (m, 2H), 2.99 (dd, J_1 = 14.9, J_2 = 5.0 Hz, 1H), 3.81 (d, J = 12.3 Hz, 1H), 4.01 (dd, J_1 = 12.3, J_2 = 3.0 Hz, 1H), 5.06-5.13 (m, 4H), 5.81-5.89 (m, 1H), 5.98-6.06 (m, 1H), 6.27 (td, J_1 = 8.0, J_2 = 1.2 Hz, 1H), 6.48-6.51 (m, 1H), 7.00 (d, J = 2.7 Hz, 1H); ^{13}C NMR (125 MHz, $CDCl_3$) δ 28.9, 31.1, 32.3, 33.2, 34.5, 41.1, 41.7, 42.8, 44.8, 44.9, 48.6, 50.4, 51.0, 54.9, 67.7, 78.2, 80.5, 116.7, 117.4, 131.6, 133.7, 135.0, 137.3 ppm;HRMS (ESI) calcd for $C_{24}H_{28}NaO_2$[M + Na] 371.1982 found 371.1983.

Synthesis of compound 19

To a suspension of sodium hydride (3 equiv) in dry DMF (5 mL), was added the compound **12** (100 mg, 0.37 mmol) and the reaction mixture was stirred for 30 min at rt. Allyl bromide

(3 equiv) was then added and stirring was continued for 12 h at the same temperature. After completion of the reaction (TLC monitoring), the reaction mixture was diluted with saturated aq. NH_4Cl (5 mL) and aqueous layer was then extracted with EtOAc (3 × 10 mL). The organic layer was washed with brine and dried over Na_2SO_4. The solvent was removed under reduced pressure and the crude product was purified by column chromatography (100–200 silica gel) using appropriate mixtures of EtOAc–petroleum ether as an eluent to afford the desired compound **19** (100 mg, 86.9%).

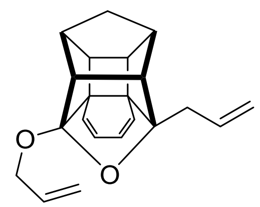

White liquid, IR (Neat) ν_{max} 3417, 3152, 2938, 1456 cm^{-1}; ^{1}H NMR (400 MHz, $CDCl_3$) δ 1.38 (d, *J* = 10.5 Hz, 1H), 1.77 (d, *J* = 10.5 Hz, 1H), 2.35-2.40 (m, 1H), 2.46 (t, *J* = 4.8 Hz, 1H), 2.54-2.59 (m, 2H), 2.68-2.72 (m, 1H), 2.74-2.78 (m, 1H), 2.86-2.92 (m, 2H), 4.20-4.33 (m, 2H), 5.09-5.18 (m, 3H), 5.27-5.33 (m, 1H), 5.45-5.49 (m, 1H), 5.58-5.61 (m, 1H), 5.78-6.01 (m, 4H); ^{13}C NMR (100 MHz, $CDCl_3$) δ 34.5, 41.9, 43.2, 44.4, 53.1, 53.6, 53.9, 54.5, 54.6, 57.6, 67.0, 93.5, 116.1, 117.9, 121.5, 122.2, 122.3, 122.9, 124.0, 133.9, 135.0 ppm; HRMS (ESI) calcd for $C_{21}H_{22}NaO_2$[M + Na] 329.1512 found 329.1516

NMR spectra of starting and final compounds

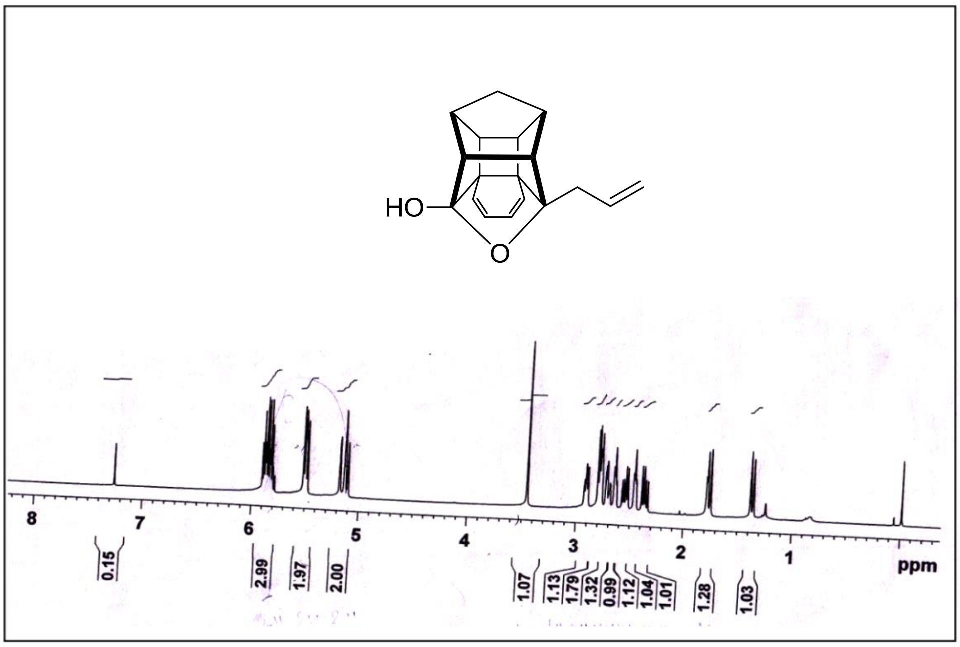

Figure 1: ^{1}H NMR of 12

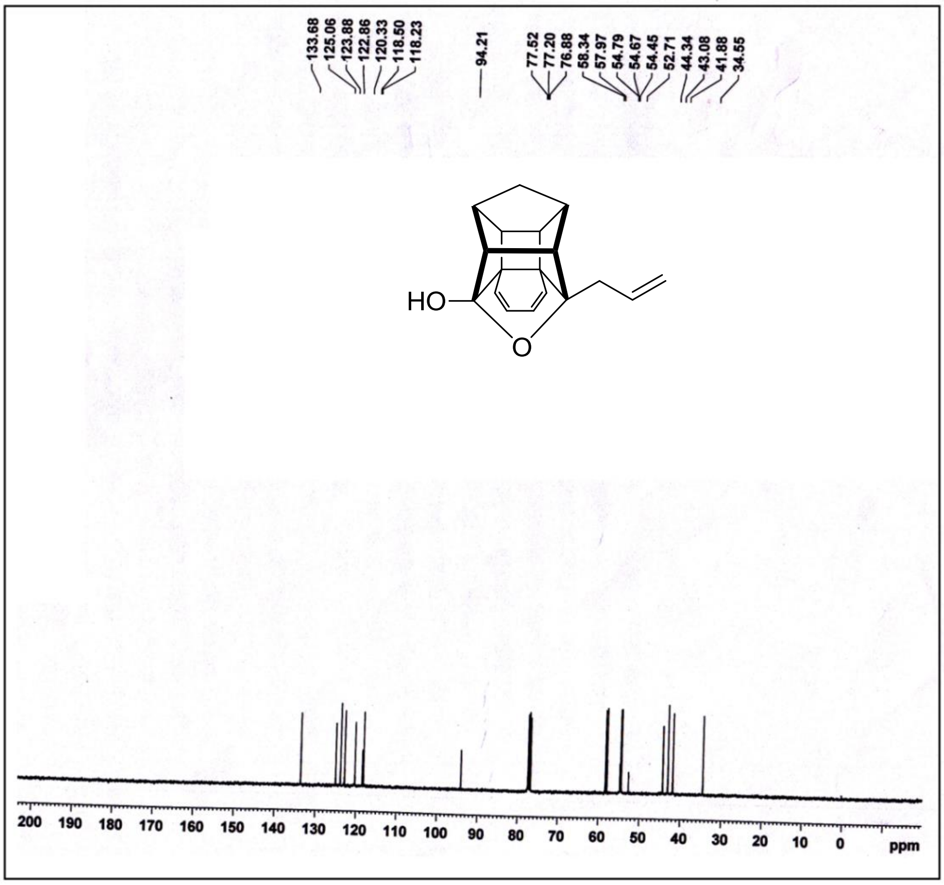

Figure 2: ^{13}C NMR of 12

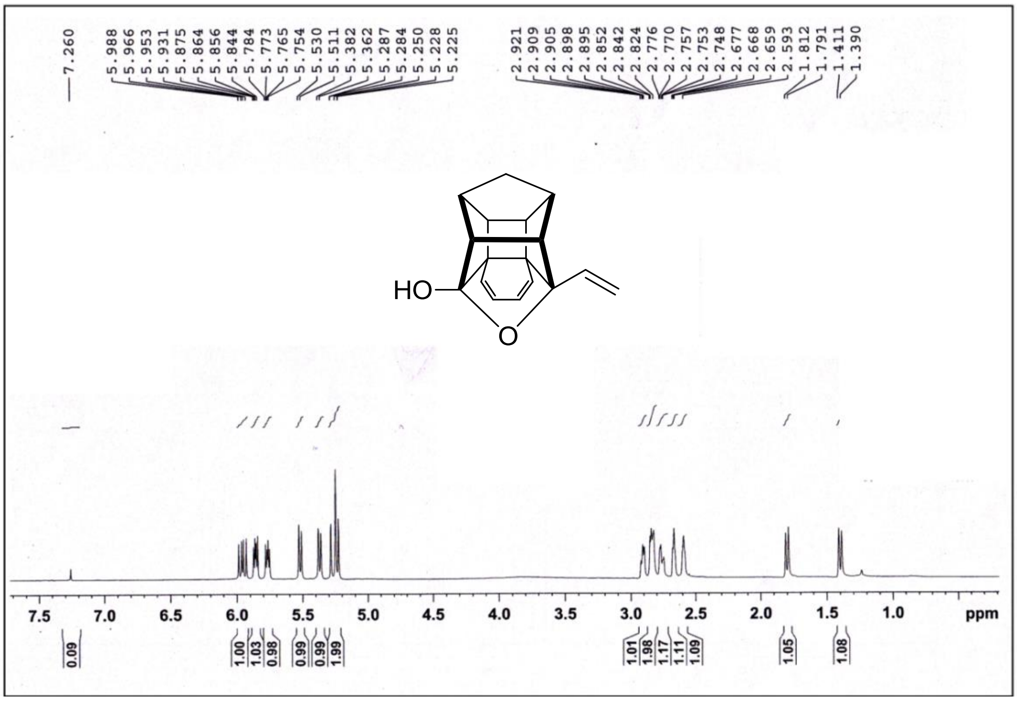

Figure 3: ^{1}H NMR of 13

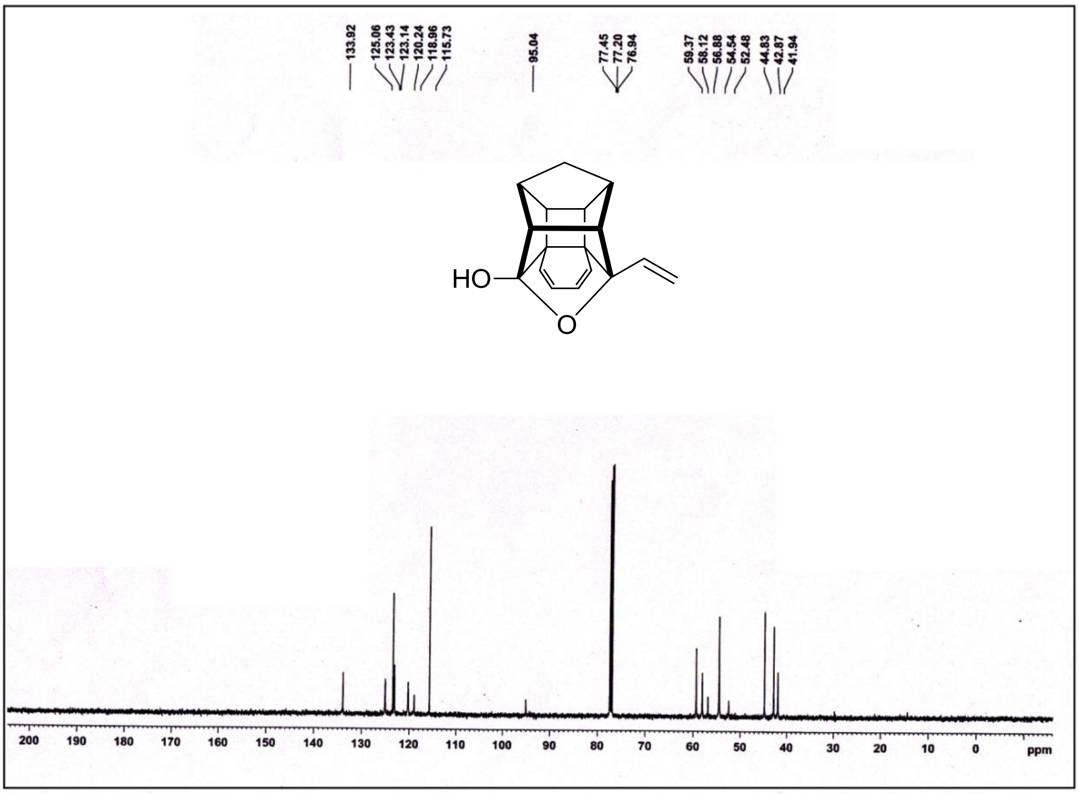

Figure 4: ^{13}C NMR of 13

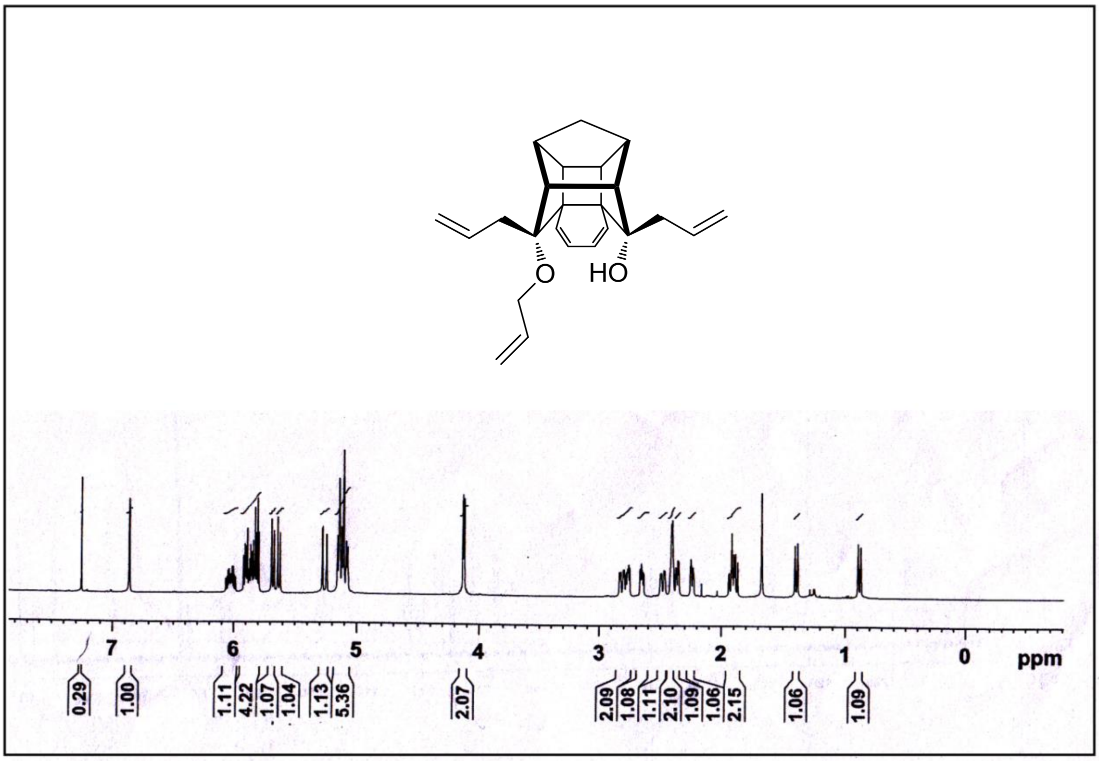

Figure 5: ^{1}H NMR of 15

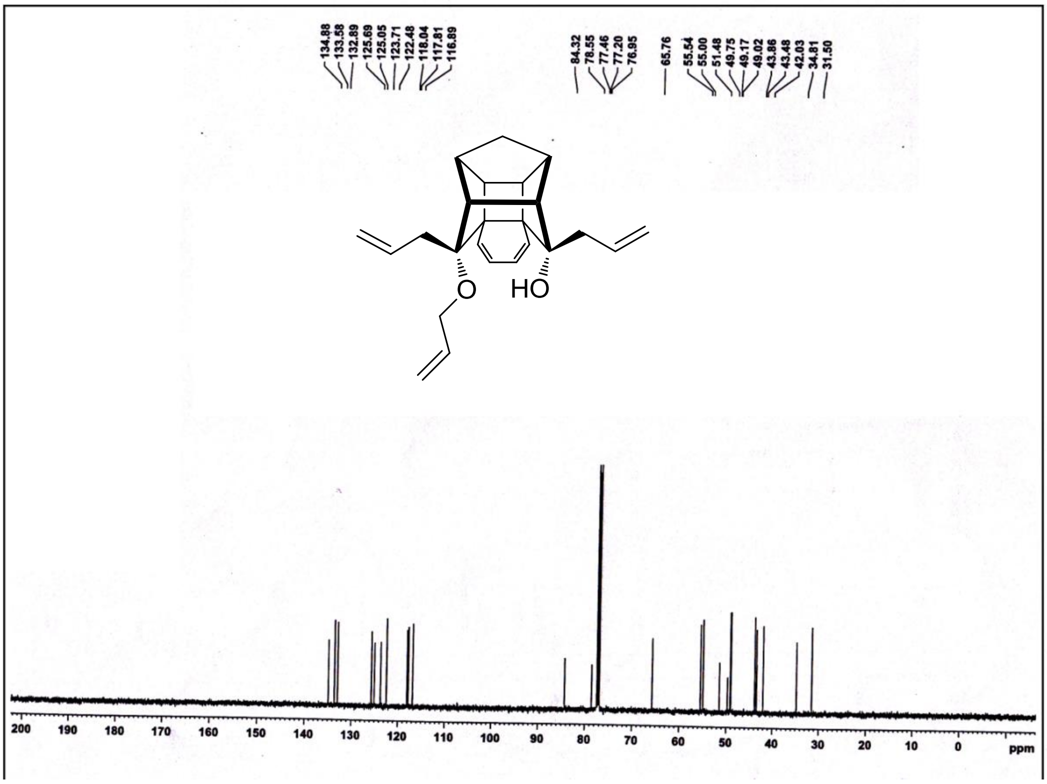

Figure 6: ^{13}C NMR of 15

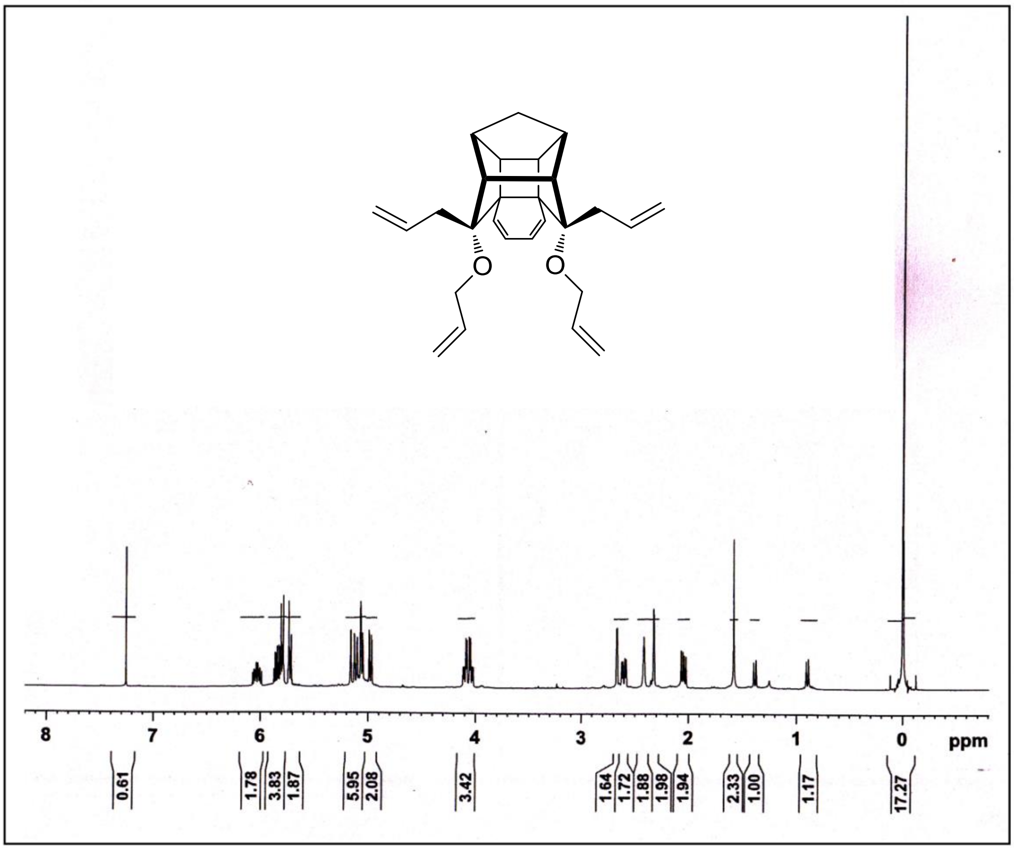

Figure 7: ^{1}H NMR of 16

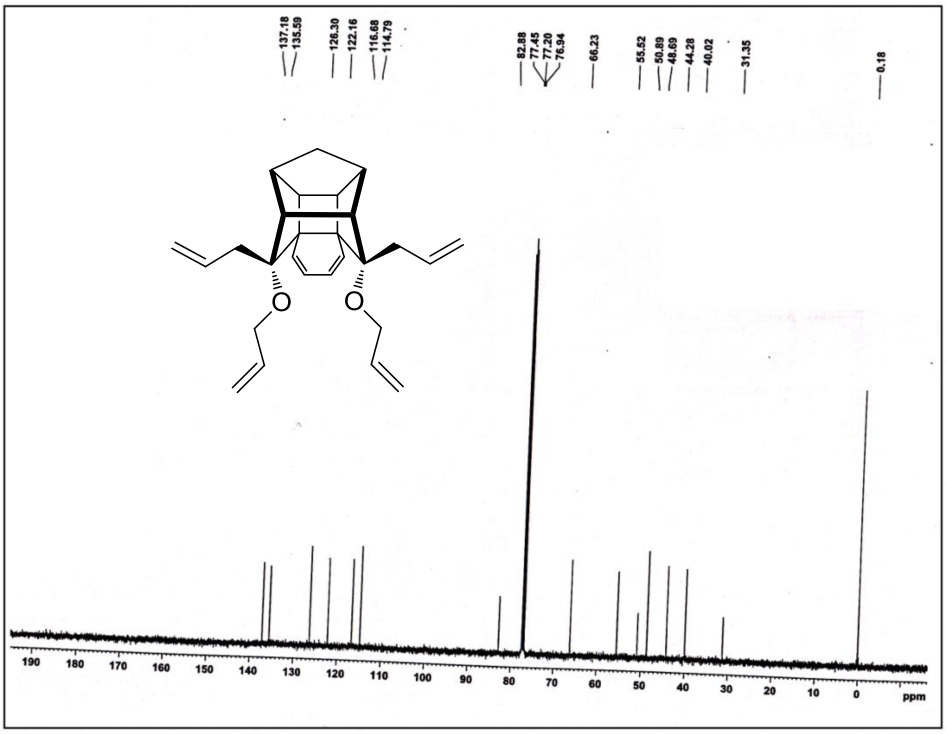

Figure 8: ^{13}C NMR of 16

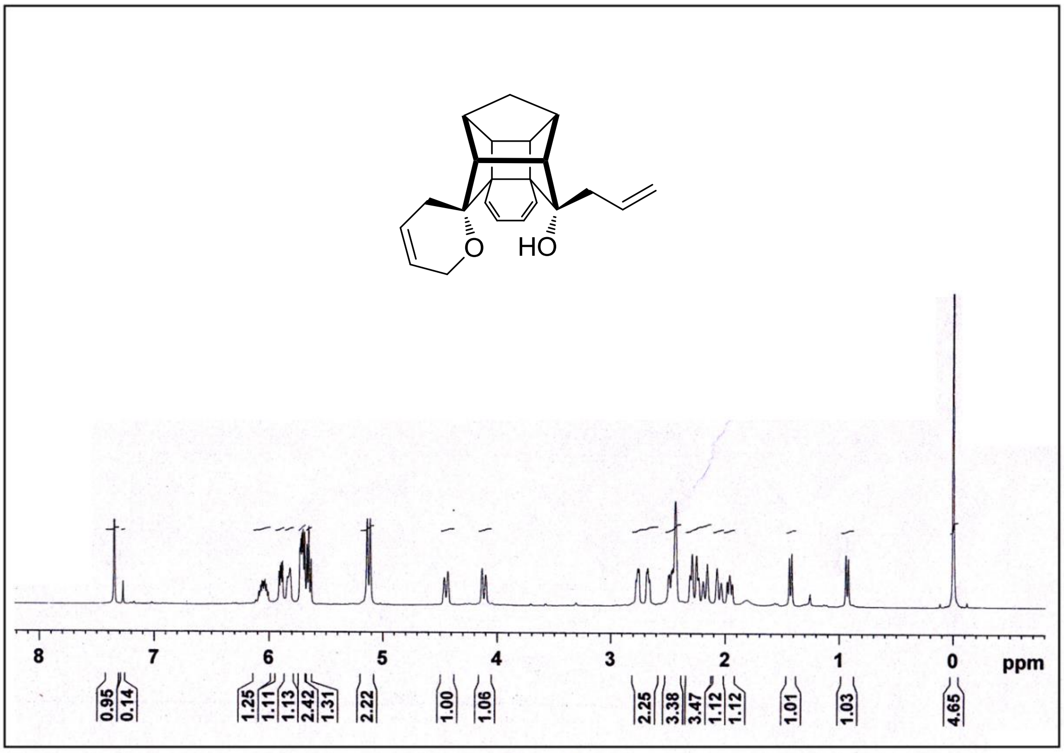

Figure 9: ^{1}H NMR of 17

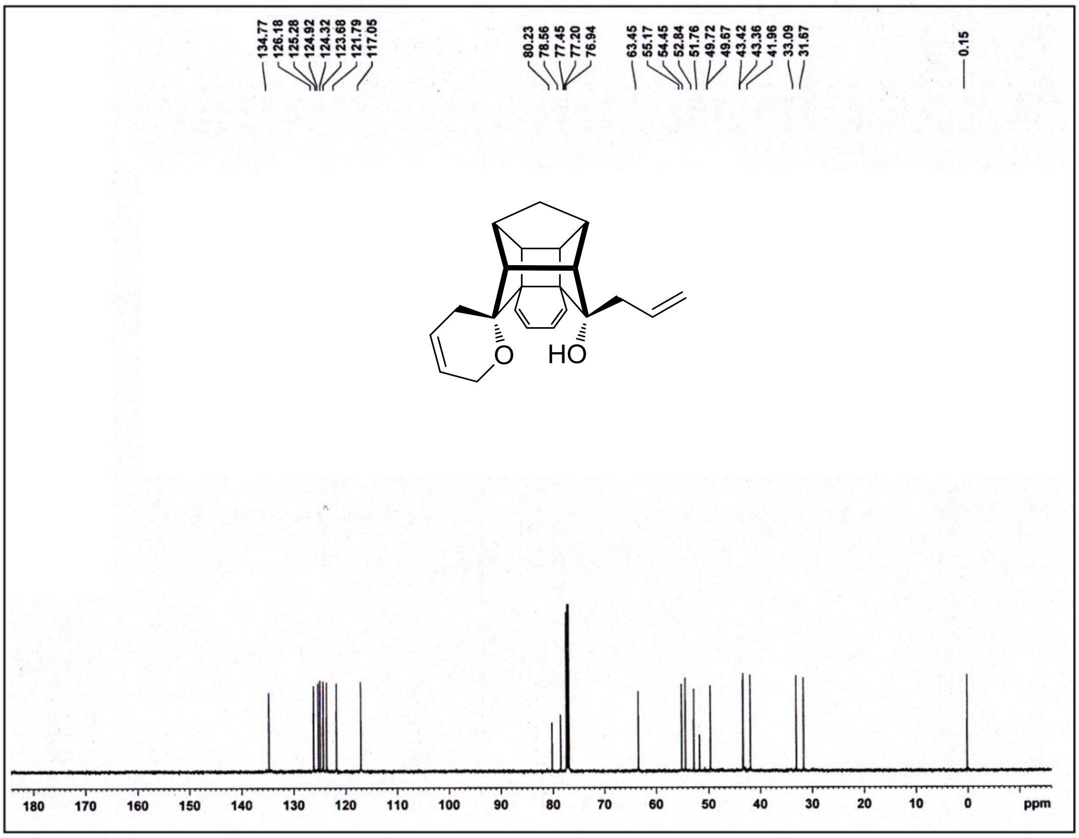

Figure 10: ^{13}C NMR of 17

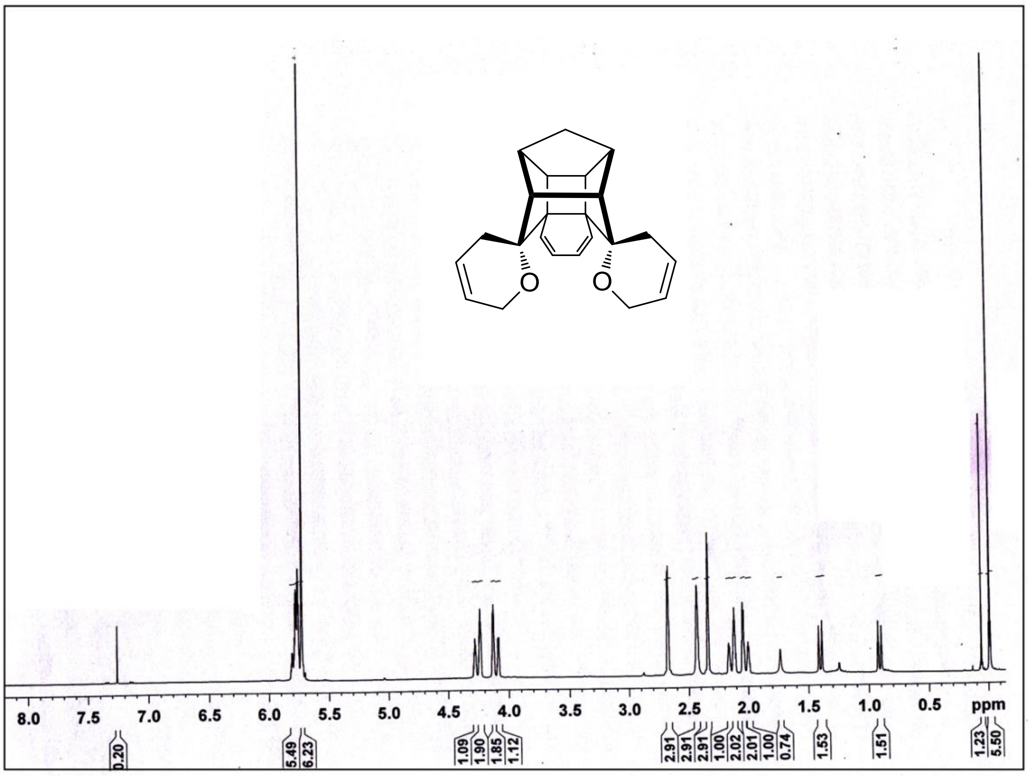

Figure 11: ^{1}H NMR of 7

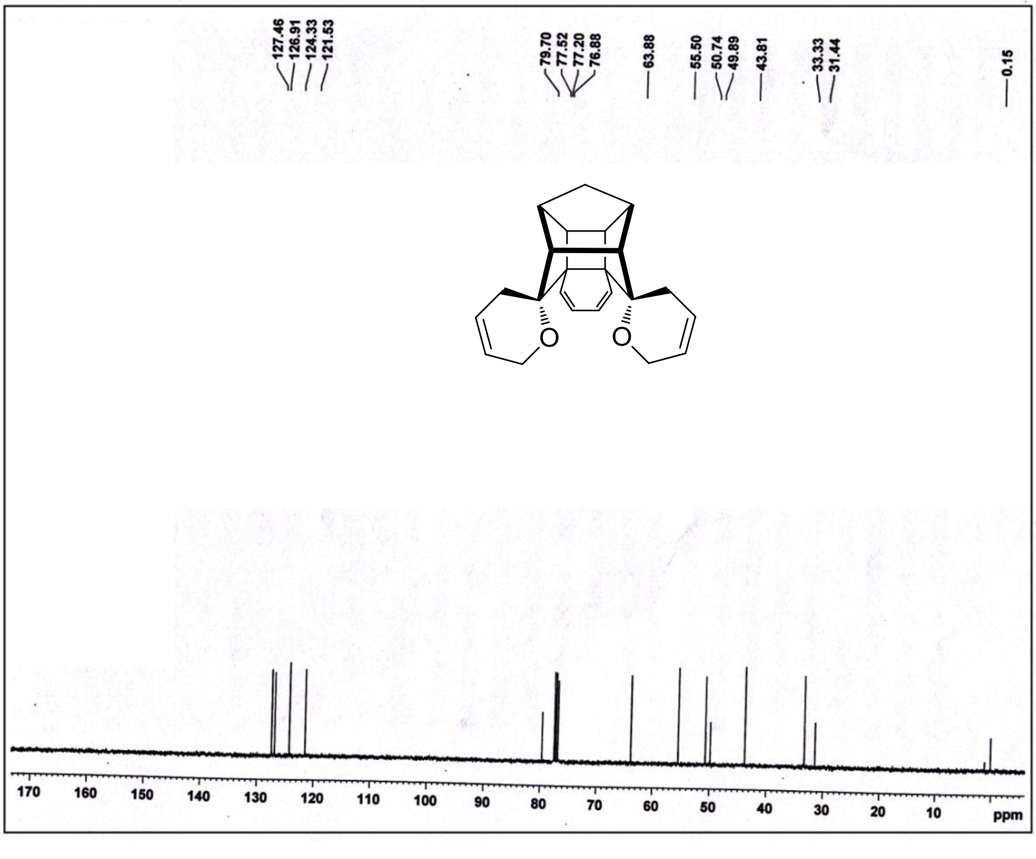

Figure 12: ^{13}C NMR of 7

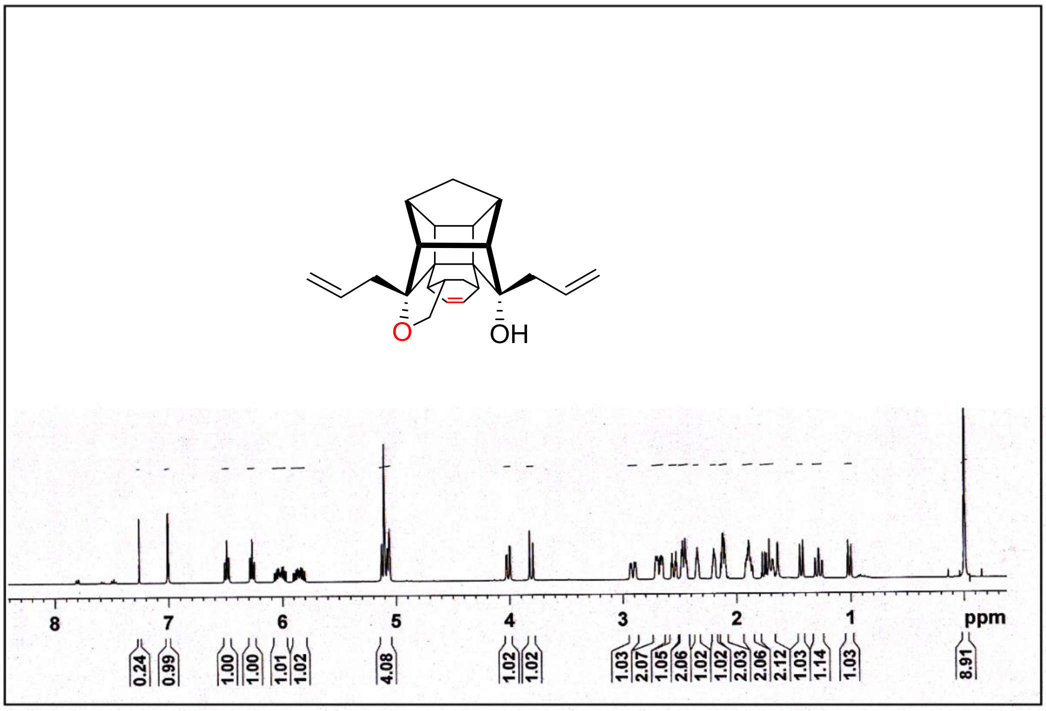

Figure 13: ^{1}H NMR of 18

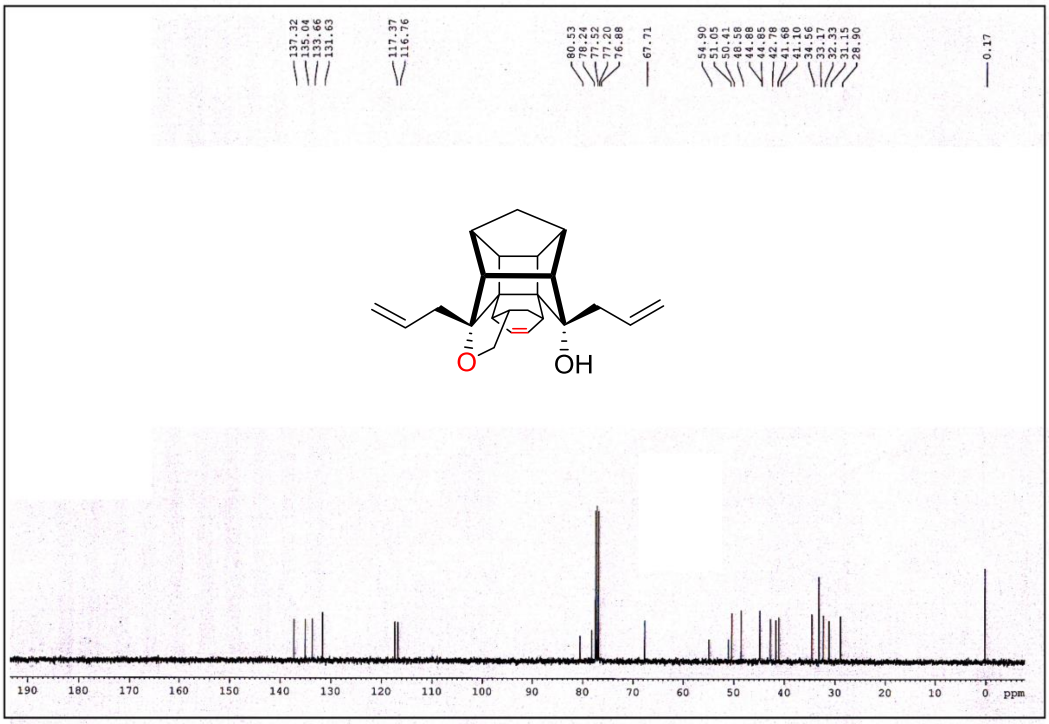

Figure 14: ^{13}C NMR of 18

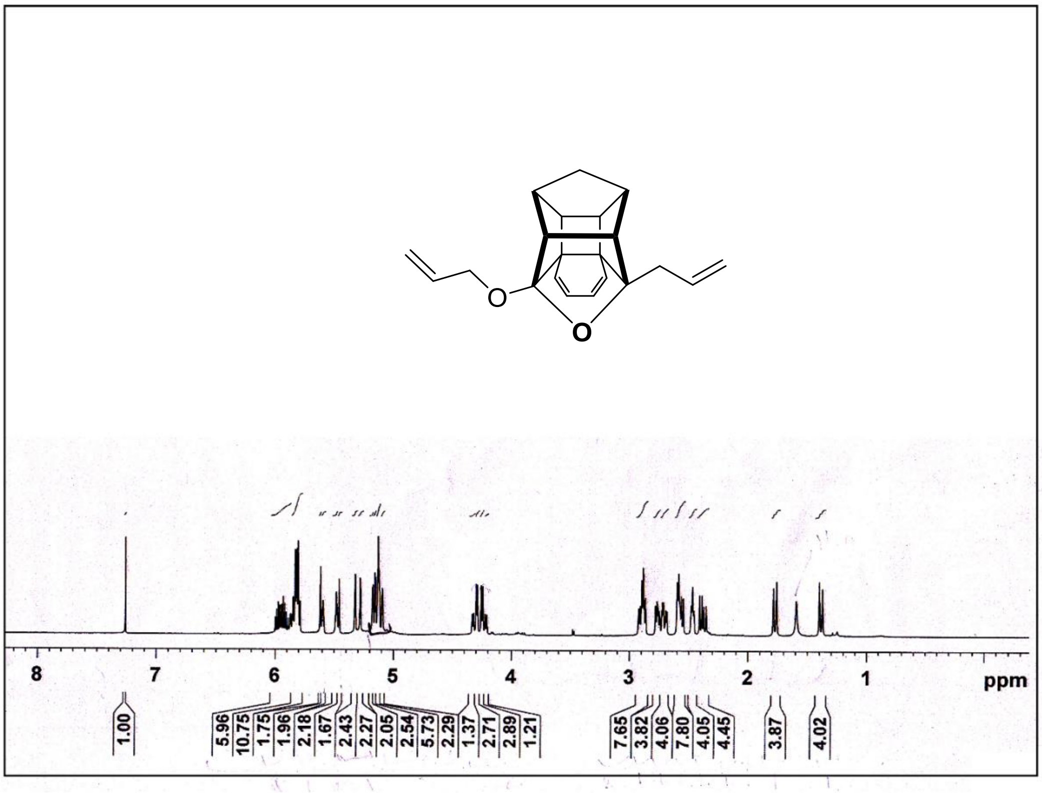

Figure 15: ^{1}H NMR of 19

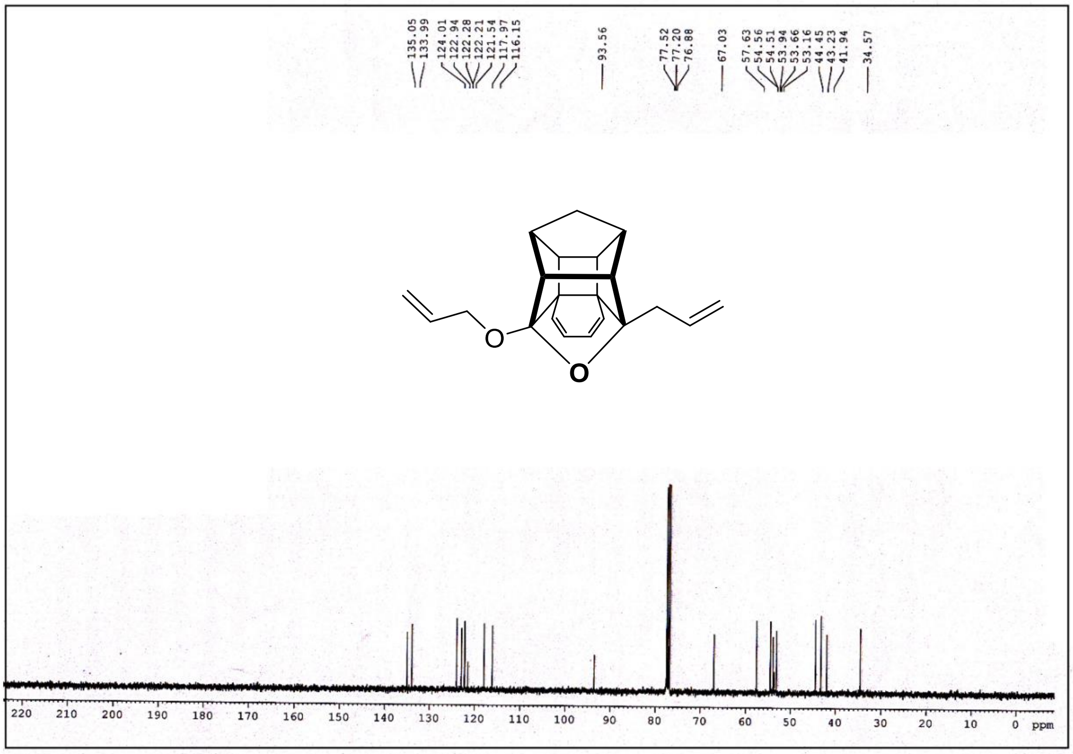

Figure 16: ^{13}C NMR of 19

X-ray crystallographic data

Bond precision: C-C = 0.0021 A Wavelength=0.71070

Cell:	a=10.929(4)	b=7.830(2)	c=16.181(5)
	alpha=90	beta=108.896(4)	gamma=90
Temperature:	100 K		

	Calculated	Reported
Volume	1310.1(7)	1310.1(8)
Space group	P 21/n	P 1 21/n 1
Hall group	-P 2yn	-P 2yn
Moiety formula	C18 H18 O2	C18 H18 O2
Sum formula	C18 H18 O2	C18 H18 O2
Mr	266.32	266.34
Dx,g cm-3	1.350	1.350
Z	4	4
Mu (mm-1)	0.086	0.086
F000	568.0	568.0
F000'	568.26	
h,k,lmax	12,9,19	12,9,19
Nref	2302	2298
Tmin,Tmax	0.963,0.991	0.966,0.991
Tmin'	0.963	

Correction method= NUMERICAL

Data completeness= 0.998 Theta(max)= 25.000

R(reflections)= 0.0439(1938) wR2(reflections)= 0.1214(2297)

S = 1.083 Npar= 181

X-ray crystallographic data and refinement parameters for compound 12 (CCDC 1053504)

Bond precision: C-C = 0.0020 A Wavelength=0.71070

Cell:	a=12.301(3)	b=10.996(3)	c=9.416(2)
	alpha=90	beta=104.243(4)	gamma=90
Temperature:	100 K		

	Calculated	Reported
Volume	1234.5(5)	1234.5(6)
Space group	P 21/c	P 1 21/c 1
Hall group	-P 2ybc	-P 2ybc
Moiety formula	C17 H16 O2	C17 H16 O2
Sum formula	C17 H16 O2	C17 H16 O2
Mr	252.30	252.31
Dx,g cm-3	1.357	1.357
Z	4	4
Mu (mm-1)	0.088	0.088
F000	536.0	536.0
F000'	536.25	
h,k,lmax	14,13,11	14,13,11
Nref	2173	2163
Tmin,Tmax	0.968,0.990	0.982,0.990
Tmin'	0.968	

Correction method= # Reported T Limits: Tmin=0.982 Tmax=0.990
AbsCorr = NUMERICAL

Data completeness= 0.995 Theta(max)= 25.000

R(reflections)= 0.0435(1896) wR2(reflections)= 0.1260(2163)

S = 1.113 Npar= 172

X-ray crystallographic data and refinement parameters for compound 13 (CCDC 1053505)

Bond precision:	C-C = 0.0020 A		Wavelength=0.71070
Cell:	a=8.0670(19)	b=10.085(2)	c=10.248(2)
	alpha=90	beta=92.849(4)	gamma=90
Temperature:	100 K		

	Calculated	Reported
Volume	832.7(3)	832.7(3)
Space group	P 21	P 1 21 1
Hall group	P 2yb	P 2yb
Moiety formula	C23 H24 O2	C23 H24 O2
Sum formula	C23 H24 O2	C23 H24 O2
Mr	332.42	332.44
Dx,g cm-3	1.326	1.326
Z	2	2
Mu (mm-1)	0.083	0.083
F000	356.0	356.0
F000'	356.15	
h,k,lmax	11,13,14	11,13,14
Nref	4506[2375]	4217
Tmin,Tmax	0.982,0.991	0.989,0.991
Tmin'	0.982	

Correction method= # Reported T Limits: Tmin=0.989 Tmax=0.991
AbsCorr = NUMERICAL

Data completeness= 1.78/0.94 Theta(max)= 29.200

R(reflections)= 0.0342(3523) wR2(reflections)= 0.0808(4217)

S = 0.997 Npar= 226

X-ray crystallographic data and refinement parameters for compound 7 (CCDC 1053506)

Bond precision:	C-C = 0.0041 A		Wavelength=1.54190
Cell:	a=25.29(2)	b=8.861(6)	c=15.870(19)
	alpha=90	beta=102.05(3)	gamma=90
Temperature:	100 K		

	Calculated	Reported
Volume	3478(6)	3478(6)
Space group	C 2/c	C 1 2/c 1
Hall group	-C 2yc	-C 2yc
Moiety formula	C24 H28 O2	C24 H28 O2
Sum formula	C24 H28 O2	C24 H28 O2
Mr	348.46	348.48
Dx,g cm-3	1.331	1.331
Z	8	8
Mu (mm-1)	0.640	0.641
F000	1504.0	1504.0
F000'	1508.09	
h,k,lmax	30,10,19	30,10,19
Nref	3199	3148
Tmin,Tmax	0.912,0.987	0.947,0.987
Tmin'	0.809	

Correction method= # Reported T Limits: Tmin=0.947 Tmax=0.987
AbsCorr = NUMERICAL

Data completeness= 0.984 Theta(max)= 68.280

R(reflections)= 0.0756(2185) wR2(reflections)= 0.2105(3148)

S = 0.941 Npar= 235

X-ray crystallographic data and refinement parameters for compound 18 (CCDC 1053507)
